# Biomechanical Properties of the Sarcolemma and Costameres of Skeletal Muscle Lacking Desmin

**DOI:** 10.3389/fphys.2021.706806

**Published:** 2021-08-19

**Authors:** Karla P. Garcia-Pelagio, Robert J. Bloch

**Affiliations:** ^1^Departamento de Fisica, Facultad de Ciencias, Universidad Nacional Autónoma de México, Mexico City, Mexico; ^2^Department of Physiology, University of Maryland School of Medicine, Baltimore, MD, United States

**Keywords:** elastimetry, intermediate filaments, myopathy, sarcomere, contractile apparatus, lateral force transmission, synemin, dystrophin

## Abstract

Intermediate filaments (IFs), composed primarily by desmin and keratins, link the myofibrils to each other, to intracellular organelles, and to the sarcolemma. There they may play an important role in transfer of contractile force from the Z-disks and M-lines of neighboring myofibrils to costameres at the membrane, across the membrane to the extracellular matrix, and ultimately to the tendon (“lateral force transmission”). We measured the elasticity of the sarcolemma and the connections it makes at costameres with the underlying contractile apparatus of individual fast twitch muscle fibers of desmin-null mice. By positioning a suction pipet to the surface of the sarcolemma and applying increasing pressure, we determined the pressure at which the sarcolemma separated from nearby sarcomeres, P_separation_, and the pressure at which the isolated sarcolemma burst, P_bursting_. We also examined the time required for the intact sarcolemma-costamere-sarcomere complex to reach equilibrium at lower pressures. All measurements showed the desmin-null fibers to have slower equilibrium times and lower P_separation_ and P_bursting_ than controls, suggesting that the sarcolemma and its costameric links to nearby contractile structures were weaker in the absence of desmin. Comparisons to earlier values determined for muscles lacking dystrophin or synemin suggest that the desmin-null phenotype is more stable than the former and less stable than the latter. Our results are consistent with the moderate myopathy seen in desmin-null muscles and support the idea that desmin contributes significantly to sarcolemmal stability and lateral force transmission.

## Introduction

Proteins of the intermediate filament (IF) superfamily in skeletal muscle are essential for the alignment of the contractile apparatus, the force it generates and how that force is transmitted, the anchoring of signaling molecules, the distribution of mitochondria, the stability of both the sarcolemma and myofibrils, cell motility, and perhaps other functions (Goldman et al., 1996; Coulombe et al., 2000; Capetanaki et al., 2007; Schopferer et al., 2009; Herrmann and Aebi, 2016; Winter et al., 2016; Klymkowsky, 2019; Eiber et al., 2020). IFs are key organizers of costameres, structures that align the cell membrane regularly with nearby myofibrils and transmit contractile force laterally from myofibrils to the extracellular matrix (Bloch and Gonzalez-Serratos, 2003; Herrmann and Aebi, 2016). These proteins fall into six structural groups (Eriksson et al., 2009; Herrmann and Aebi, 2016; Etienne-Manneville, 2018). Types 1 and 2 are keratins, which in mature skeletal muscle are represented primarily by Ker8, Ker18, and Ker19 (Ursitti et al., 2004; Stone et al., 2007; Lovering et al., 2011; Shah et al., 2012; Muriel et al., 2020) form heteropolymeric filaments. The type 3 filament protein of mature muscle is desmin, which homopolymerizes but can also associate with the type 4 protein synemin (Bellin et al., 1999; Mizuno et al., 2001, 2004). Type 5 proteins, the neurofilaments, are not known to be expressed in muscle. Type 6 proteins, the lamins, are restricted to the nuclear envelope.

Diseases of skeletal muscle linked to IFs have been associated with, among others, mutations in desmin (“desminopathies”), and the lamins (Emery-Dreifuss muscular dystrophy, LMNA-related congenital muscular dystrophy; Capetanaki et al., 2007; Nigro and Savarese, 2014; Smogavec et al., 2017).

Many studies of the role of the IF proteins in muscle have examined the effects of eliminating the expression of one or more of them by homologous recombination. We have used this approach to test the roles of Ker19, desmin, and synemin in the structure and function of murine muscle (O’Neill et al., 2002; Ursitti et al., 2004; Stone et al., 2007; Lovering et al., 2011; Shah et al., 2012; García-Pelagio et al., 2015; Muriel et al., 2020). All three proteins are present in structures that surround the Z-disk of the contractile apparatus and in IFs that approach the cytoplasmic surface of the sarcolemma at costameres (Bloch et al., 2002; O’Neill et al., 2002; Ursitti et al., 2004; Lovering et al., 2011; Shah et al., 2012; Muriel et al., 2020). The absence of desmin or Ker19 alters the organization of costameres, and measurements by elastimetry show that the absence of synemin weakens the links between the costameres and underlying sarcomeres (García-Pelagio et al., 2015).

Desmin links the Z-disks of adjacent myofibrils to each other and to the sarcolemma, as well as to the mitochondria and nucleus. It provides structural stability to the sarcomere and serves as a scaffold to stabilize the myofiber (O’Neill et al., 2002; Capetanaki et al., 2007). It is the most prominent IF protein in mature muscle, and its absence has more profound effects on muscle than the absence of either Ker19 or synemin (Sam et al., 2000; O’Neill et al., 2002; Shah et al., 2004; Ursitti et al., 2004; Meyer and Lieber, 2012; Meyer et al., 2013; Muriel et al., 2020). Here, we report the result of elastimetry studies that demonstrate desmin’s prominent role in stabilizing the sarcolemma and its linkage to the contractile apparatus of skeletal muscle. We also compared these results to our previous results studying dystrophin and synemin (García-Pelagio et al., 2011, 2015).

## Materials and Methods

### Animals

Female and male age-matched (8–12 weeks) control mice and mice null for desmin (des −/−), for synemin (syn −/−) or for dystrophin (*mdx*) were used. A total of 64 adult mice [*N* = 10 per “knockout” (KO) group, and *N* = 8 per control group] were studied. FVB mice were purchased from Taconic, Hudson, NY, and des −/− mice (in the FVB and 129SVJ backgrounds; Milner et al., 1996) were raised in the Central Animal Facility of the University of Maryland, Baltimore. C57Bl/10ScSn and *mdx* (C57Bl/10ScSn-DMD-*mdx*) mice were purchased from The Jackson Laboratory (Bar Harbor, ME). C57Bl/6 and syn −/− mice, in the C57Bl/6 background, were raised in the Animal Facility of the University of Maryland, Baltimore (García-Pelagio et al., 2015). Before removal of muscles and dissection of myofibers from the extensor digitorum longus (EDL) muscle, animals were anesthetized with isoflurane (2.5%, with an oxygen flow rate of 0.8 L/min at 14.7 psi, 21°C) and euthanized by cervical dislocation. We did not observe any differences due to sex in the few experiments in which we had sufficient numbers of each to compare. All of the experiments were in accordance with the Institutional Animal Care Committee of the University of Maryland School of Medicine.

### Biomechanical Properties

**Figure 1:**
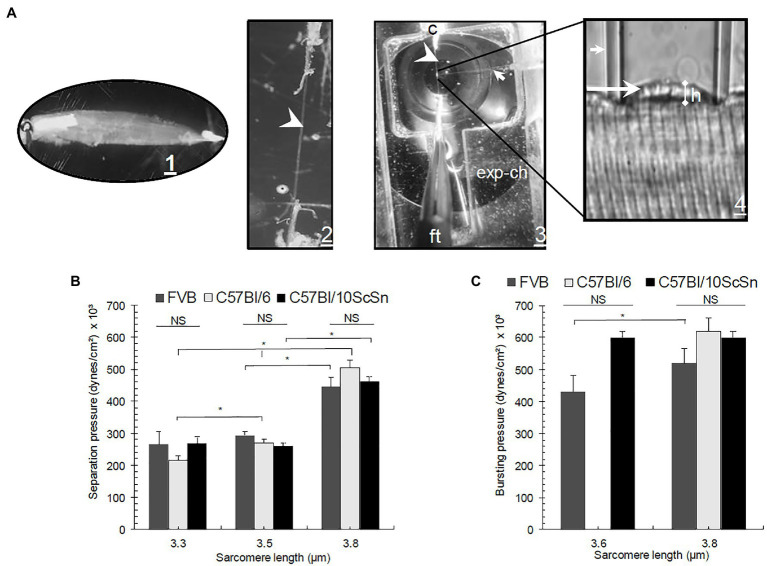
**(A1)** Experimental procedure for elastimetry measurements. Whole extensor digitorum longus (EDL) is removed with its tendons and placed in Kreb’s solution (in mM:135 NaCl, 5 KCl, 1 MgCl_2_, 15 NaHCO_3_, 11 glucose, 1 Na_2_HPO_4_, and 2.5 CaCl_2_, equilibrated with 95% O_2_ and 5% CO_2_, pH 7.0). **(A2)** The muscle is reduced by teasing to a single isolated fiber in relaxing solution, still attached to its tendons (arrowhead). **(A3)** The fiber is placed in an experimental chamber (exp-ch), where sarcomere length (SL) is fixed with a clip (c) and fine tweezers (ft). **(A4)** A borosilicate pipet (small arrow) is placed on the surface of the fiber and negative pressure is applied manually with a Hg manometer attached to a four-key valve to set P inside the syringe, inducing the formation of a bleb (long arrow) of variable height (h). **(B)** P_separation_ and **(C)** P_bursting_ graphs in three different control strains: FVB, C57Bl/6, and C57Bl/10ScSn as a function of SL. Bars indicate mean ± SD. ^*^Significant difference within the same control strains at different SL. NS, no significant difference. Statistical analysis was performed with a one-way ANOVA using a Tukey *post hoc* test.

Sarcolemmal separation and sarcolemmal bursting pressure were measured with elastimetry, as described (Rapoport, 1972; García-Pelagio et al., 2011). A single fiber attached to its tendons was obtained by placing a small bundle of EDL fibers in relaxing solution (in mM:185 K acetate, 2.5 Mg acetate, 10 imidazole propionate, 2.5 Na_2_ATP, and 5 EGTA pH 7.1; Figures 1A1, 2). A mechanically isolated fiber was transferred to an experimental chamber (exp-ch) set at 8 ± 1°C with a Peltier system and placed under a microscope (E. Leica Microsystems, Wetzlar, Germany; Figure 1A3). The sarcomere length (SL) was set by stretching the fiber by its tendons with a micromanipulator (Narishige M-2 Labtron Scientific Products, Farmingdale, NY). Longitudinal strain is generated when the SL is increased to >3 μm. The SL is fixed by pulling on the tendons with micromanipulators. A large-bore suction micropipette with an internal opening of 0.50–0.66 times the diameter of the fiber was placed on the surface of the teased fiber and suction pressure (P) from 14 to 670 × 10^3^ dyne/cm^2^ was applied through a U-shape Hg manometer. Suction pressures were adjusted for the strain studied. P exerted to the outer surface of the membrane was calculated from P = ρg*h* [dynes/cm^2^], where *ρ* = 13.5 g/cm^3^, *g* = 9.81 m/s^2^, and *h* is the difference of levels in the U-manometer relative to P = 0 (cm). The deformation of the sarcolemma and myofibrils relates to the elastic behavior of the distortion and tension lines in response to suction pressure, P, to generate the P-h curves.

**Figure 2:**
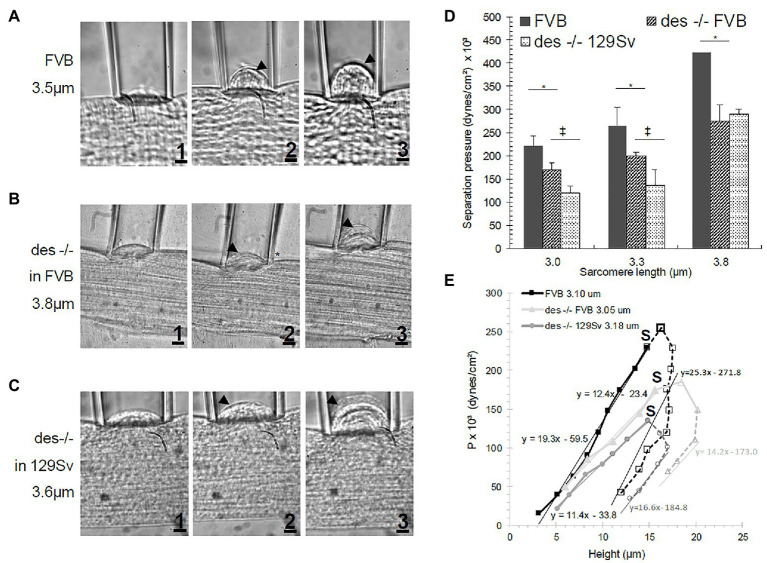
Sarcolemmal bleb increasing in size with increasing suction pressure (1-3) in **(A)** FVB, **(B)** des −/− FVB or **(C)** 129Sv muscle fibers. Arrowhead represents the separation of the sarcolemma from the cytoskeleton. **(D)** Effect of SL on separation pressure in FVB and des −/− mice. Bars indicate mean ± SD. ^*^Significant difference between des −/− FVB and its control strain at the same SL. ^‡^Significant difference comparing both des −/− mutants in different background strains. Statistical analysis was performed with a one-way ANOVA with a Tukey post hoc test. **(E)** P-h curve in FVB and des −/− (in FVB and 129Sv background) myofibers. Filled symbols and solid lines represent data before separation of the sarcolemma from the contractile elements. Open symbols and segmented lines represent data after separation. S denotes P_separation_. Regression lines equations are given for each strain. P_separation_ for des −/− was smaller than for WT, indicating a weaker attachment of the sarcolemma to underlying contractile elements.

Three regions can be readily observed in the P-h curves. In the first, at low negative pressures, the sarcolemma with the underlying contractile apparatus forms a small bubble (or “bleb”) of variable height (h, in μm) within the pipet (see Figure 1A4). The sarcolemma remained closely associated with nearby myofibrils under the bleb. The P-h values obtained in this segment are reversible, i.e., as long as the system is intact, the same pressure will generate the same height whether pressures are increased from lower to higher values, or decreased from higher to lower values (not shown). In the next segment of the P-h curve, as pressures reach the maximum that the intact system can sustain, the sarcolemma separates physically from the underlying contractile apparatus (S in Figure 2E), and the pressures needed to maintain the height of the bleb decrease in comparison to the first region of the P-h curve, in some instances after a small additional increase in pressure. In this segment, the sarcolemma distended more for the same increase in pressure. Only minimal increases in h, if any, can be elicited by further increasing the pressure. The pressure at which separation occurs, or P_separation_, is the first of the P values we determine to compare fibers of different phenotypes (Rapoport, 1972; García-Pelagio et al., 2011). In the third segment, a gradual reversal of the pressure in the pipet reveals the ability of the sarcolemma to relax back to its original shape. This region of the curve is also reversible, but larger changes in h occurred for the same changes in P. Thus, hysteresis in the P-h curve indicates that the biomechanical system differs before and after separation of the sarcolemma.

If, rather than a decrease in pressure, it is increased further, the sarcolemmal bleb continues to expand until it bursts, at what we refer to as the “bursting pressure,” or P_bursting_. This is the second of the P values we determined to compare different phenotypes (Rapoport, 1972; García-Pelagio et al., 2011). As we described in earlier publications (García-Pelagio et al., 2011, 2015), these methods *per se* can only be applied at sarcomere lengths somewhat longer than normal (3–4 μm, compared to 2 μm in relaxed muscle) to avoid the fiber being completely sucked into the pipet, which would eliminate any possibility of studying the biomechanical properties of the sarcolemma (García-Pelagio et al., 2020).

Changes in the deformation of the sarcolemma correlate to the height of the bleb, which was acquired with a 10.2 Mpix digital camera (Sony α-330, Tokyo, Japan) and analyzed with Image J (National Institute of Health, Bethesda, MD).

### Stabilization of the Membrane

Constant pressure of 80 × 10^3^ dyne/cm^2^ was applied to the membrane to determine the time course over which a bleb formed and reached a stable height. After analyzing the images, h-t curves were obtained in which time (t) was measured in minutes and the height of the bleb (h) in μm. The h-t curves resemble an RC charging circuit and follows an exponential curve. In our case, the curve is represented by h(t) = h (1–e^–t/τ^), from which the time constant (τ) can be calculated (García-Pelagio et al., 2008). This experimental analysis can determine how rapidly the cell surface responds to a specific applied pressure.

### Statistics

All values are reported as mean ± SD. Statistical significance was assessed with a one-way ANOVA. We performed a Tukey *post hoc* analysis to determine significant differences between mutant groups. Significance was set at *p* < 0.05.

## Results

We have used elastimetry to determine the deformability of the sarcolemma, the stability of its attachments to the underlying contractile apparatus, and its ability to resist bursting at high pressures. As described in Rapoport (1972) and García-Pelagio et al. (2011), we use a large bore suction pipet applied to the surface of an isolated myofiber to apply negative pressure, drawing a portion of the sarcolemma and nearby myofibrils into the pipet. When P is increased, the height of the “bubble” in the pipet increases (Figures 2A1,B1,C1). At higher values of P, designated as P_separation_, the sarcolemma separates from the underlying contractile apparatus (e.g., arrowheads from Figures 2A2,3,B2,3,C2,3) presumably because the attachments between the peripheral myofibrils and the sarcolemma were lost.

We plotted the pressures needed to induce changes in the height of the bleb pulled into the pipet, both before and after separation of the sarcolemma from the contractile apparatus. As shown in Figure 2E, these curves appear as tilted, inverted U shapes. As we reported earlier (García-Pelagio et al., 2011, 2015), myopathic muscle fibers show a shallower curve than controls (represented by the regression lines fit by the equation y = mx + b), with specific increases in height induced by lower applied pressures. In all cases, hysteresis occurs after separation (Figure 2E segmented lines; S denotes the point of separation), as greater heights of the bleb can be maintained by lower applied pressures. We observed these results for the desmin-null as well. Notably, the curve for the desmin-null in the 129SV background was shallower than in the FVB background, so the FVB control is the more appropriate comparison. The results show that, as expected, the FVB control has a steeper U-shaped curve (continuous black line in Figure 2E) than the desmin-null FVB (continuous light gray line in Figure 2E), consistent with lower pressures being required for generating blebs both before and after separation.

We applied this approach to desmin-null muscle fibers in two genetic backgrounds, 129SV and FVB, and compared the results to control muscle fibers in the FVB, C57Bl/6J, and C57Bl/10ScSn backgrounds (Figures 1B,C). As expected, the values for P_separation_ (Figure 1B) and P_bursting_ (Figure 1C), we determined for the three control strains did not vary significantly over a range of SL. Consistent with previous experiments (García-Pelagio et al., 2011), we found that P_separation_ is a function of SL, i.e., as SL increases, higher pressures are needed to separate the membrane from nearby myofibrils. Sarcomeres can be stretched to 3.8 μm without losing the interactions between thin and thick filaments. In particular, we observed up to a 2-fold (*p* < 0.05) difference in P_separation_ when we increased the SL from 3.3 to 3.8 μm in the same control strains (Figure 1B).

We show examples of the distensibility of the sarcolemma at low pressures in Figures 2A–C and quantitative measurements in Figure 2D. Figures 2A1,B1,C1 show the initial distension of control and desmin-null sarcolemma that occurs at low pressures. Figures 2A2,B2,C2 show separation of the sarcolemma from the contractile apparatus, while Figures 2A3,B3,C3 show further distension of the sarcolemma as pressures are further increased. The results, quantitated in Figure 2D, revealed that the desmin-null sarcolemma has a significantly lower P_separation_ (*p* < 0.05) than controls at sarcomere lengths greater or equal to 3.0 μm. As the two controls, we examined, 129SV and FVB, differed in their hysteresis curves (Figure 2E, light and dark segmented gray lines), we limited our direct comparisons of desmin-nulls in the FVB background and control mice to SL in which both were measured in mice of the FVB background. The results at sarcomere lengths of 3.0, 3.3, and 3.8 μm showed clear differences in P_separation_ of 24, 25, and 38%, respectively (*p* < 0.05; see Figure 2D). This suggests that the stability of sarcolemmal links to the underlying contractile apparatus is significantly reduced in desmin-null muscle fibers and has a strong dependence on sarcomere length.

**Figure 3:**
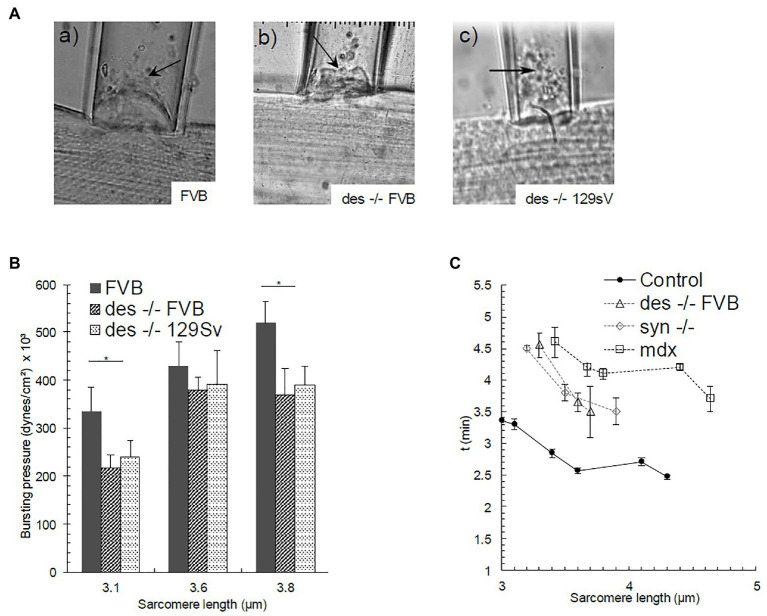
Bursting of the sarcolemmal bleb in **(Aa)** FVB, and **(Ab)**, **(Ac)** des −/− strains (SL = 3.5, 3.8, and 3.6 μm respectively). Bursting is denoted with an arrow. **(B)** P_bursting_ as a function of SL. Bars indicate mean ± SD. ^*^Significant difference between des −/− FVB and its control strain at the same SL. Statistical analysis was performed with a one-way ANOVA. **(C)** Time needed for the bleb to reach a steady-state as a function of SL in Control FVB, des −/− (FVB), syn −/−, and *mdx* myofibers. Bars indicate mean ± SE.

In a number of experiments, we continued to increase the pressures on the blebs until the membranes burst, to measure P_bursting_ (Figure 3A). The height of the underlying contractile structures after bursting varied from 4 to 9 μm depending on the mutant or control strain. As observed with other myopathic muscle fibers (García-Pelagio et al., 2011, 2015), the bursting pressures we measured for desmin-null FVB fibers stretched to sarcomere lengths of 3.1 and 3.8 μm were significantly lower than those for FVB controls (1.5 and 1.4-fold, respectively; Figure 3B). Values for desmin-null 129SV fibers were similarly reduced compared to control FVBs at these same sarcomere lengths and tended to be lower than controls at the other sarcomere lengths we examined. These results suggest that the sarcolemma, once isolated from underlying contractile structures, is less stable in desmin-null myofibers than in controls, and that some desmin remains associated with the sarcolemma after separation occurs.

We also examined the time required for the bleb to reach a stable height as a function of sarcomere length, at pressures below P_separation_. The formation of the bleb does not occur immediately, but some minutes are instead needed to achieve a stable height. This time delay indicates that the sarcolemma-costameres-myofibril complex acts as a viscoelastic system. We noticed in our earliest studies that control fibers stabilized faster than those in syn −/− and *mdx* myofibers (Figure 3C; García-Pelagio et al., 2008), consistent with their higher stiffness. We found the same to be true for desmin-null FVB fibers at sarcomere lengths below 5 μm, i.e., SLs at which the sarcolemma-costamere-myofibril complex in desmin-null fibers was more elastic than that in control FVB fibers.

Finally, we compared the results, we obtained with desmin-null muscle to those we reported earlier for synemin-null and *mdx* (dystrophin-null) muscle (Table 1; García-Pelagio et al., 2011, 2015). We limited our comparisons to sarcomere lengths of 3.3, 3.5, and 3.8 μm, as these were held in common in all three studies, shown as averaged values for all three SLs in Table 1. Although, the mutants were studied in different background strains, the differences of P_separation_ among the three were significantly different (Table 1; *p* < 0.05). P_separation_ for desmin-null muscle was less than that for synemin-null fibers but greater than that for *mdx*, suggesting that the links between the costameres and nearby sarcomeres were stronger in the desmin-null than in the *mdx*, but weaker than those in the synemin-null. P_bursting_ for the desmin-null was also intermediate between the *mdx* and synemin-null, suggesting that the intrinsic stability of the sarcolemma, once separated from the underlying contractile apparatus, was weaker in the desmin-null than in the synemin-null, but stronger than in the *mdx*. The P_bursting_ of the isolated sarcolemma in desmin-null fibers was significantly less than that in controls, suggesting that the stability of the sarcolemma is reduced in desmin-null fibers. A possible explanation is that the costameres in desmin null muscle are less disrupted than in dystrophin-null but more than in synemin-null muscle, leaving less support for the isolated membrane and thus lower P_bursting_.

**Table 1:** Average of P_sep_ and P_bursting_ expressed as mean ± SD from all SLs examined.

	FVB	C57Bl/6	C57Bl/10ScSn	des −/− FVB	des −/− 129Sv	syn −/−	*mdx*
P_sep_ × 10^3^ (dynes/cm^2^)	336.2 ± 46.1	360.0 ± 16.3	346.7 ± 37.4	220.1 ± 18.9^*^^‡^	207.7 ± 12.6	300.0 ± 17.0^*^^‡^	180.3 ± 33.1^*^^‡^
P_bursting_ × 10^3^ (dynes/cm^2^)	521.6 ± 35.7	620.1 ± 42.0	631.7 ± 47.0	375.4 ± 40.7^*^	401.7 ± 34.6	480.3 ± 35.0^*^^†^	373.6 ± 34.6^*^^†^

* Significant difference compared to its respective control strain (desmin null FVB to FVB; synemin null to C57Bl/6, and mdx to C57Bl/10ScSn).

† Significant difference between mdx and synemin null strains.

‡ Significant difference between desmin null FVB, synemin null, and mdx strains.

In addition, the time to the formation of stable blebs in desmin null muscle was less or equal to that in the synemin null muscle but greater than in the *mdx* (Figure 3C), suggesting that the viscoelasticity of the sarcolemma-costamere-myofibril system in the desmin null is also intermediate between these genotypes.

## Discussion

Desmin-null muscle has been reported by several laboratories to be morphologically, biomechanically, and functionally abnormal, exhibiting myofiber branching, altered nuclear and myofibrillar structures, a loss of costameres at the sarcolemma, increased deformation by passive loads, altered signaling cascades, lower contractile strength, reduced isometric tension in fast and slow muscle and more rapid fatigue (Milner et al., 1996; Li et al., 1997; Wieneke et al., 2000; Shah et al., 2001, 2004, 2012; Balogh et al., 2003; Kreplak et al., 2008; Lovering et al., 2011; Goodall et al., 2012; Heffler et al., 2020) reviewed by Capetanaki et al. (2007) and Agnetti et al. (2021). Several observations have also suggested a role for desmin in coupling the contractile apparatus to the sarcolemma, including the dispersal of costameric proteins when desmin is absent, as well as an increased gap between the sarcolemma and the nearest myofibrils and a decrease in the membrane strain in desmin-null fibers (O’Neill et al., 2002; Shah et al., 2004; Lovering et al., 2011). Here, we use elastimetry methods, originally designed by Rapoport (1972) and modified in our laboratory (García-Pelagio et al., 2011), to examine the stability of the sarcolemma and its links to nearby sarcomeres directly. Elastimetry applied to the surface of a myofiber measures the distensibility of the sarcolemma while it is linked to the underlying contractile apparatus, its distensibility once the pressures applied have severed those links, and the ability of the sarcolemma to resist bursting at even higher pressures. By all three measures, the sarcolemma of desmin-null myofibers and its links to nearby contractile structures are less stable than those of controls.

A longitudinal strain is introduced when the SL is fixed at lengths ≥ 3 μm, thereby stabilizing the contractile structures. As the sarcolemma and associated contractile apparatus are drawn into the pipet by the applied suction pressure, a lateral strain is exerted. These two structures, combined with their links to nearby myofibrils, account for the mechanical properties we measure when the system is intact. It is therefore reasonable that increased longitudinal strain will create increased resistance to deformation by the suction pipet, and that higher suction pressures will be required to yield the same changes in the height of the bleb. Our observations suggest that the contractile apparatus contributes significantly to the stiffness of the sarcolemma before separation occurs, consistent with the weakened sarcolemmal links to the myofibrils in KO muscles.

There is considerable evidence that the presence of desmin filaments increases the number of sarcomeres (Shah et al., 2004). This results in an increase in the stiffness of the myoplasm, which deforms less upon longitudinal strain than in the absence of desmin (Shah et al., 2012). Consistent with this, aggregates of desmin filaments formed by mutant desmins increase resistance to deformation (Sam et al., 2000). Only a few studies address the role of desmin in stabilizing the sarcolemma, however. Our own observations showed that costameres were disrupted in most fast twitch fibers of the desmin-null mouse (O’Neill et al., 2002). Likewise, studies of Cauchy stress as a function of SL in isolated fibers demonstrated a decrease in membrane longitudinal stress in the absence of desmin, congruent with a decrease in sarcomere longitudinal strain and suggesting a “continuity between the sarcolemma and the myofibrillar lattice” (Shah et al., 2004). To the best of our knowledge, the results reported here are the first to quantitate the contributions of desmin to the stability of the sarcolemma and its links through costameres to nearby sarcomeres. We suggest that the determinants from membrane stability (before separation occurs at increased pressures) are determined by the system composed of the sarcolemma, costameres, and nearby myofibrils, consistent with the results cited above.

Although, the effects of increasing suction pressure on the sarcolemma and its links to the underlying contractile apparatus were described nearly 50 years ago by Rapoport (1972), the structural changes that occur with increasing pressure are still poorly understood. Visualization by phase microscopy indicates that the sarcolemma, costameres and nearby sarcomeres act as a single system at low pressures, but that at a pressure, S, the two structures separate and afterward act independently of each other. Separation is not likely to be complete, however, as in many studies we observe further increases in h as a function of P that follow a slope similar to, or slightly less than, the slope we observed at lower pressures. This suggests that some elements of the intact system are retained at the sarcolemma even after separation. These may include cytoskeletal elements on the cytoplasmic surface, collagen or other extracellular proteins on the extracellular surface, or other, less specific interactions (e.g., van der Waals, electrostatic forces) with membrane-adjacent macromolecules (Carton et al., 1962; Elliot, 1967; O’Neill et al., 2002; Hanft et al., 2006). Further studies of the sarcolemma isolated by the suction technique have the potential of revealing the nature of these elements and how they influence sarcolemmal elasticity.

The phenotype of the desmin-null mice and those of the *mdx* and synemin-null mice, studied earlier, are consistent with the severity of the myopathies seen in these animals. As summarized in Table 1, both P_separation_ and P_bursting_ in the desmin-null were higher than in the *mdx* and lower than in the synemin-null. The *mdx* mouse shows greater levels of muscle degeneration and regeneration, is somewhat weaker and is significantly more susceptible to injury than the desmin-null (Capogrosso et al., 2017; Delacroix et al., 2018). This supports the idea that dystrophin stabilizes the sarcolemmal membrane, independent of its contributions at costameres and their links to nearby myofibrils. Synemin-nulls show no evidence of weakness or degeneration/regeneration, although they appear to be more susceptible to injury than desmin-nulls (Lovering et al., 2011; García-Pelagio et al., 2015). Perhaps most notably, the costameres in these three mutant strains differ significantly. The costameres of *mdx* mice are highly abnormal and, leave large membrane regions without cytoskeletal support (Williams and Bloch, 1999). By contrast, although the costameres of the desmin-null are disrupted, the sarcolemma retains much of its support (O’Neill et al., 2002; Ferry et al., 2020). Costameres in the synemin-null are similar to controls (García-Pelagio et al., 2015). Consistent with these observations, earlier immunohistological studies showed that muscle fibers lacking desmin retained dystrophin at the sarcolemma but lacked synemin at the regions of the sarcolemma overlying Z-disks and at the Z-disks within myofibrils (O’Neill et al., 2002; Lovering et al., 2011; Kerr, 2012). Synemin null fibers retained desmin and dystrophin at the sarcolemma and desmin at the Z-disks (Kerr, 2012; García-Pelagio et al., 2015); and fibers lacking dystrophin retain desmin and syntrophin at both the sarcolemma and at Z-disks (O’Neill et al., 2002; Lovering et al., 2011). Thus, the biomechanical changes at the sarcolemma of these three mutant strains roughly parallel the physiological and morphological changes we have documented.

Although our results indicate that desmin plays an important role at the sarcolemma as well as in the myoplasm, we cannot rule out the possibility that some of the changes we have measured are indirect, perhaps caused by changes in signaling or gene expression that are associated with the presence of desmin (Russell et al., 2006; Heffler et al., 2020). We focused our studies on young mice, to avoid the additional effects of aging, which can lead to increased fibrosis in desmin null and control mice (Faulkner et al., 1990, 2007; Meyer and Lieber, 2012; Hughes et al., 2015) but other, potentially compensatory changes may accompany the absence of desmin as mice develop and mature.

We have proposed (Bloch and Gonzalez-Serratos, 2003; Bloch et al., 2004) that a significant amount of force generated by skeletal muscle is transmitted laterally, from the contractile apparatus, through intermediate filaments (as well as through actin; Tidball, 1991; Sonnemann et al., 2006) to the sarcolemma *via* costameres, across the sarcolemma to the extracellular matrix, and through the matrix to the tendon. Evidence supporting this model has been reported by Street (1983), the Faulkner laboratory (Ramaswamy et al., 2011), and others (Monti et al., 1999; Passerieux et al., 2007; Dowling et al., 2021). Here, we show that the stability and viscoelastic properties of the sarcolemma and its association with underlying sarcomeres are compromised in the absence of desmin. Desmin-null muscles are also ~30% weaker than controls. Thus, our results are consistent with the idea that lateral force transmission is mediated at least in part by desmin filaments.

## Data Availability Statement

The original contributions presented in the study are included in the article/supplementary material, further inquiries can be directed to the corresponding author.

## Ethics Statement

The animal study was reviewed and approved by Institutional Animal Care Committee of the University of Maryland School of Medicine.

## Author Contributions

All authors listed have made a substantial, direct and intellectual contribution to the work, and approved it for publication.

## Conflict of Interest

The authors declare that the research was conducted in the absence of any commercial or financial relationships that could be construed as a potential conflict of interest.

## Publisher’s Note

All claims expressed in this article are solely those of the authors and do not necessarily represent those of their affiliated organizations, or those of the publisher, the editors and the reviewers. Any product that may be evaluated in this article, or claim that may be made by its manufacturer, is not guaranteed or endorsed by the publisher.
